# Functional Analysis of Aquaporin Water Permeability Using an *Escherichia coli*-Based Cell-Free Protein Synthesis System

**DOI:** 10.3389/fbioe.2020.01000

**Published:** 2020-08-19

**Authors:** Ke Yue, Jihong Jiang, Peng Zhang, Lei Kai

**Affiliations:** The Key Laboratory of Biotechnology for Medicinal Plants of Jiangsu Province, School of Life Sciences, Jiangsu Normal University, Xuzhou, China

**Keywords:** aquaporin, cell-free protein synthesis, water permeability, detergent, proteoliposomes

## Abstract

Aquaporins are essential water channel proteins found in all kingdoms of life. Although the water permeability of aquaporins has been well characterized, sample preparation for aquaporin water permeability assays remains challenging and time-consuming. Besides the difficulty in overexpressing membrane proteins in a cell-based expression system, the unique requirement for homogeneity in aquaporin proteoliposome sample preparations for water transport assays further increases the complexity. In this study, a complementary Cell-free Protein Synthesis (CFPS) method is described in detail, providing three different strategies for the preparation of aquaporin proteoliposome samples. Aquaporin can be produced either as a pellet fraction and then resolubilized, or co-translationally as a detergent-soluble fraction. Furthermore, aquaporin can be directly incorporated into liposomes, which was included in the CFPS reactions. Although proteoliposomes tend to fuse during the incubation of the CFPS reactions, an additional treatment of the fused samples with detergent, followed by a detergent removal step, can re-form homogenously sized proteoliposomes suitable for functional analysis. Using this method, we successfully characterized aquaporins from both prokaryotic and eukaryotic organisms. In particular, in the presence of liposomes, the developed CFPS expression system is a fast and convenient method for sample preparation for the functional analysis of aquaporins.

## Introduction

Aquaporins are integral membrane proteins that facilitate the rapid and selective movement of water and neutral low-molecular-mass solutes across biological membranes along osmotic gradients. Although the function and structure of aquaporins have been extensively characterized (Preston et al., 1992; Agre et al., 1993; Verkman and Mitra, 2000; Tornroth-Horsefield et al., 2006), sample preparation for the functional analysis of aquaporins is still challenging and time-consuming (Yue et al., 2019). Three main methods are commonly used to determine the water permeability of aquaporins: (i) The *Xenopus laevis* oocyte system. First, the cDNA encoding target aquaporins is injected and overexpressed in the native oocyte membrane. Water permeability is then calculated as the volume change of the oocytes under an osmotic shock, which is recorded *via* a light microscope (Preston et al., 1992; Yang and Verkman, 1997). (ii) The yeast protoplast system (Pettersson et al., 2006). Here, the water permeability of protoplasts overexpressing target aquaporins is measured using stopped-flow spectrophotometry (Bertl and Kaldenhoff, 2007). (iii) The liposome system, where overexpressed target aquaporins are reconstituted into artificial liposomes, and the permeability of the resulting proteoliposomes is determined according to the changed intensity of scattered light at a fixed angle *via* stopped-flow spectrophotometry under osmotic shock (Ye and Verkman, 1989; Zeidel et al., 1992). The first two methods do not require isolation and purification of aquaporins; however, they suffer from the influence of endogenous integral membrane proteins (Ho et al., 2009). Although the liposome system offers a precise measurement of the permeability of specific aquaporins, the sample preparation process is still laborious and challenging, especially when aquaporins are overexpressed *in vivo* (Yue et al., 2019). In contrast, the Cell-free Protein Synthesis (CFPS) system, which is devoid of living cells and cell membrane barriers, has the unique advantage of being an open system (Henrich et al., 2015; Rues et al., 2016), allowing the introduction of various additives in a co-translational manner (Schwarz et al., 2007, 2008). In particular, hydrophobic reagents such as detergents and lipids can be introduced directly into the CFPS system to promote the correct folding of newly expressed membrane proteins (Junge et al., 2011; Roos et al., 2013).

During the last few years, we have developed a set of protocols for the production and functional characterization of aquaporins based on the CFPS system (Kai et al., 2010; Kai and Kaldenhoff, 2014; Yue et al., 2019). Here, we first detail a number of detergents suitable for soluble aquaporin expression. Secondly, we provide a detailed purification strategy, including detergent exchange on a column for the downstream reconstitution process. Furthermore, based on the work of Hovijitra et al. (2009), we describe a detailed protocol for the direct insertion of cell-free (CF) expressed aquaporins into liposomes using a modified procedure that is suitable for the continuous exchange cell-free (CECF) expression mode (Kai and Kaldenhoff, 2014; Yue et al., 2019). In this study, we have summarized our previous methods and provided a systematic protocol for the expression and functional characterization of aquaporins based on an *Escherichia coli* CFPS system, including template design, aquaporin expression and purification, proteoliposome preparation, and an aquaporin water permeability assay using stopped-flow spectrophotometry.

## Materials and Equipment

### Materials

#### Materials for the CFPS Reaction

**FIGURE 1:**
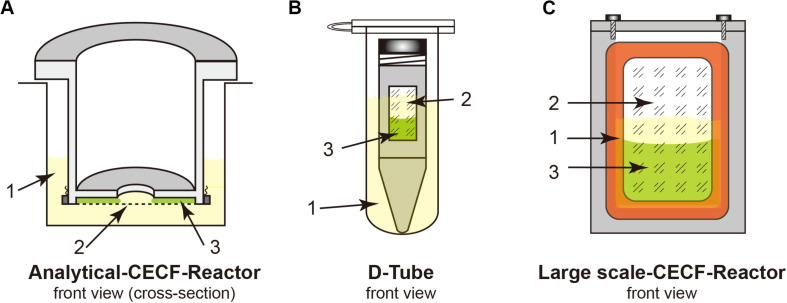
Cell-free Protein Synthesis (CFPS) reactors for the continuous exchange cell-free (CECF) configuration. Panels **(A,C)** were custom made from Plexiglas. **(B)** Commercial D-Tube dialyzer either in a 2-mL Eppendorf tube or in 15–50-mL Falcon tubes, depending on the size of the D-Tube dialyzer. 1, light yellow indicates the outside feeding mixture (FM); 2, dialysis membrane; 3, light green indicates the reaction mixture (RM).

1. 50 × Complete^®^ Protease Inhibitor Cocktail tablets (Roche Diagnostics), 1 tablet/mL of Milli-Q water.
2. Amino acid mixtures containing 8 mM each of the 20 natural amino acids (weigh in all the compounds and dissolve the powders in Milli-Q water; the stock remains turbid).
3. RCWMDE mix containing 16.7 mM each amino acid (the stock remains turbid).
4. 1 M acetyl phosphate lithium potassium salt (AcP) (Sigma–Aldrich), adjusted to pH 7.0 with KOH.
5. 1 M phospho(enol)pyruvic acid monopotassium salt (PEP) (Sigma–Aldrich), adjusted to pH 7.0 with KOH.
6. An NTP mixture containing 90 mM ATP, 60 mM CTP, 60 mM GTP, and 60 mM UTP (Sigma–Aldrich), adjusted to pH 7.0 with NaOH.
7. Pyruvate kinase (Roche Diagnostics), 10 mg/mL.
8. RiboLock^®^ RNAse Inhibitor (Fermentas), 40 U/μL.
9. Total *E. coli* tRNA (Roche Diagnostics), 40 mg/mL.
10. Folinic acid (as calcium salt), 10 mg/mL (Sigma–Aldrich).
11. Polyethylene glycol 8000 (PEG 8000) (Sigma–Aldrich), 40% (*w*/*v*).
12. 4 M potassium acetate (KOAc).
13. 2.4 M Hepes/20 mM EDTA, pH 8.0 adjusted with KOH.
14. 500 mM 1,4-dithiothreitol (DTT).
15. *Escherichia coli* S30 extract, stored frozen at −80°C (see section “Preparation of S30 Extract and T7RNAP”).
16. T7 RNA polymerase (T7RNAP), stored frozen at −80°C (see section “Preparation of S30 Extract and T7RNAP”).
17. Template DNA (plasmid DNA or linear PCR products), 200–500 ng/μL [see section “CFPS Reactions (Overnight)”].
18. Reaction container: analytical and preparative scale reaction container [see Figure 1 and section “Protein Purification (3 h)”]; D-tube containers, 12–14 kDa MWCO (Merck Biosciences); Slide-A-Lyzer, 10 kDa MWCO (Pierce); dialysis membrane tubing, 12–14 kDa MWCO (Spectra/Por^®^2).

19. Plasmid and PCR product purification kits (Qiagen, Macherey and Nagel).

#### Materials for S30 Extract and T7RNAP Preparation

1. 40 × S30-A/B buffer: 400 mM Tris-acetate pH 8.2; 560 mM Mg(OAc)_2_; 2.4 M KCl.

Supplement 1 × S30-A buffer with 6 mM β-mercaptoethanol. Supplement 1 × S30-B buffer with 1 mM DTT and 1 mM phenylmethylsulfonyl fluoride (PMSF).

2. 40 × S30-C buffer: 400 mM Tris-acetate pH 8.2; 560 mM Mg(OAc)_2_; 2.4 M KOAc.

Supplement 1 × S30-C buffer with 0.5 mM DTT.

3. 2 × YTPG medium: 22 mM KH_2_PO_4_, 40 mM K2HPO4, 100 mM glucose, tryptone 16 g/L, yeast extract 10 g/L, NaCl 5 g/L.
4. LB medium: Peptone 10 g/L, yeast extract 5 g/L, NaCl 5 g/L.
5. Buffer T7RNAP-A: 30 mM Tris-HCl pH 8.0, 50 mM NaCl, 10 mM EDTA, 10 mM β-mercaptoethanol, 5% glycerol.
6. Buffer T7RNAP-B: 30 mM Tris-HCl pH 8.0, 50 mM NaCl, 1 mM EDTA, 10 mM β-mercaptoethanol, 5% glycerol.
7. Buffer T7RNAP-C: 30 mM Tris-HCl pH 8.0, 1 M NaCl, 1 mM EDTA, 10 mM β-mercaptoethanol, 5% glycerol.
8. Buffer T7RNAP-D: 10 mM K_2_HPO_4_/KH_2_PO_4_ pH 8.0, 10 mM NaCl, 0.5 mM EDTA, 1 mM DTT, 5% glycerol.
9. 20% streptomycin sulfate.
10. *Escherichia coli* strain for extract preparation: A19 (*E. coli* Genetic Stock Center, New Haven, CT, United States).
11. BL21(DE3) Star transformed with pAR1219 for T7RNAP preparation (Li et al., 1999).

#### Detergents and Lipids

1. Detergents: Triton X-100 (Merck Biosciences); Brij^®^35, Brij^®^58, Brij^®^S20, digitonin, and tyloxapol (Sigma; LMPG {1-myristoyl-2-hydroxy-sn-glycero-3-(phospho-rac-[1-glycerol])} and LPPG {1-palmitoyl-2-hydroxy-sn-glycero-3-(phospho-rac-[1-glycerol])} (Avanti Polar Lipids); Fos-Choline-12 (dodecylphosphocholine) and Fos- Choline-16 (n-hexadecylphosphocholine) (Affymetrix^®^ Anatrace); and DDM (*N*-dodecyl-β-D-maltoside) and β-OG (octyl-β-D-glucopyranoside) (AppliChem).
2. Lipids: *E. coli* polar lipids (Avanti Polar Lipids) and L-α-phosphatidylcholine from soybean (Sigma–Aldrich).

#### Materials for Purification

1. Buffer P-B (binding buffer): 20 mM Tris-HCl pH 7.8, 150 mM NaCl, 20 mM imidazole, 0.05% DDM.
2. Buffer P-W (washing buffer): 20 mM Tris-HCl pH 7.8, 150 mM NaCl, 80 mM imidazole, 0.05% DDM.
3. Buffer P-E (elution buffer): 20 mM Tris-HCl pH 7.8, 150 mM NaCl, 300 mM imidazole, 0.05% DDM.

#### Materials for the Water Permeability Assay

1. 10 × Buffer R (reconstitution): 1 M MOPS buffer, pH 7.5.
2. Hyperosmotic solution: Buffer R + 400 mM sucrose.
3. Whatman polycarbonate membrane filter (200 nm) and filter supports (Florham Park, NJ, United States).
4. SM-2 beads (Bio-Rad, München, Germany).

#### Immunoblotting Assay

1. Penta⋅His Antibody, BSA-free (QIAGEN, Hilden, Germany).
2. Horseradish peroxidase (HRP)-conjugated goat anti-mouse IgG (Sigma–Aldrich, Taufkirchen, Germany).
3. Polyvinylidene difluoride (PVDF) membrane (Merck Millipore, Darmstadt, Germany).

### Equipment

1. Fermenter for 5–10 L of culture volume (B. Braun Biotech).
2. French press or other high-pressure, cell-disruption equipment.
3. Photometer.
4. Centrifuges and a set of rotors (Sorvall or Kontron).
5. Dialysis tubing, 30 kDa MWCO (Spectrum).
6. Mini extruder (Avanti Polar Lipids).
7. Ultrasonic water bath.
8. Thermo-shaker for incubation.
9. Chromatographic system (GE Healthcare).
10. Q-Sepharose column.
11. Ultrafiltration devices, 30 kDa MWCO.
12. Ultracentrifuge and suitable rotors (Beckman Coulter).
13. Rotary evaporator with vacuum control.
14. Stopped-flow spectrophotometer (SFM 300, Bio-Logic SAS, Claix, France).
15. ZetaPlus particle sizing software 2.27 for dynamic light scattering particle size analysis.
16. Gel electrophoresis system and blotting system.
17. Gel imaging system.

## Methods

### Preparation of S30 Extract and T7RNAP

#### Preparation of S30 Extract (2.5 Days)

S30 is the key component of CFPS reactions, providing all the enzymes needed for translation and energy regeneration. A number of detailed protocols have been published elsewhere (Kai et al., 2012, 2015). Here, we briefly described the major steps:

1. Cell fermentation (using 2 × YTPG medium) at 37°C with vigorous aeration until the cells reach the mid-log growth phase, followed by fast chilling to 18°C.
2. Cell washing (using S30-A buffer) followed by cell lysis (in 110% S30-B buffer) using a French press (one passage above 1,000 psi is sufficient to disrupt the cells) or similar high-pressure cell homogenizer.
3. Centrifugation at 30,000 × *g* (S30) to clarify the lysate.
4. Runoff steps with high salt and heat (42°C) to remove endogenous mRNA and undesired proteins.
5. Overnight dialysis with one exchange against S30-C buffer and another S30 centrifugation step.
6. Aliquot the resulting supernatant, freeze with liquid nitrogen, and store at −80°C until use. Approximately 60 mL of S30 extract can be obtained from 10 L of fermented *E. coli* cells.

#### T7RNAP Preparation (1 Day)

Overexpression of T7RNAP is performed through transforming *E. coli* BL21 (DE3) Star cells with the pAR1219 plasmid (Li et al., 1999). One step of anion exchange chromatography followed by a concentration step is sufficient to obtain a highly functional enzyme. The resulting T7RNAP should show a prominent band around the 90 kDa region with Coomassie Blue staining following SDS–PAGE. The average yield is approximately 20,000–40,000 units per liter of culture.

1. Inoculate a fresh, overnight culture of newly transformed BL21(DE3) Star cells containing the pAR1219 plasmid into fresh LB medium at a 1:100 ratio. Let the cells grow at 37°C with shaking and induce with 1 mM IPTG when the OD600 reaches 0.6–0.8. Incubate for a further 5 h and harvest by centrifugation at 8,000 × *g* for 15 min at 4°C.
2. Resuspend the cell pellet in 30 mL of T7RNAP-A buffer and disrupt the cells by passaging once through a French press at 1,000 psi. Remove the cell debris by centrifugation at 20,000 × *g* for 30 min at 4°C. The ensuing steps should be performed at 4°C.
3. Adjust the supernatant to a final concentration of 4% streptomycin sulfate by the stepwise addition of a 20% stock solution with gently mixing to remove the released DNA. Incubate on ice for 5 min and centrifuge at 20,000 × *g* for 30 min at 4°C.
4. Perform anion exchange purification by loading the supernatant onto a 40-mL Q-Sepharose column equilibrated with T7RNAP-B buffer and wash the column extensively with T7RNAP-B buffer.
5. Elute the T7RNAP with a 50–500 mM NaCl gradient using T7RNAP-C buffer for 10 column volumes at a flow rate of 3–4 mL/min. Collect the fractions and analyze aliquots by SDS–PAGE (overexpression of T7RNAP should be indicated by the presence of a prominent band at approximately 90 kDa; however, significant impurities might still exist).
6. Pool the T7RNAP-containing fractions (combine only peak fractions) and dialyze against T7RNAP-D buffer overnight. Adjust to a final concentration of 10% glycerol and concentrate the resulting T7RNAP-containing fraction to a total protein concentration of 3–4 mg/mL by ultrafiltration (T7RNAP begins to precipitate at higher concentrations). Adjust to a final concentration of 50% glycerol, aliquot, and store at −80°C.

### DNA Template Preparation (0.5–1 Day)

Clone the aquaporin gene of interest between the T7 promoter and T7 terminator. Common vectors such as pET (Merck Biosciences) or pIVEX (Roche Diagnostic) are frequently used. Templates for CFPS reactions should be prepared using standard commercial ‘Midi’ and ‘Maxi’ plasmid kits. Mini kits are usually not suitable due to the low quality of the obtained purified DNA. The resulting DNA should be dissolved in Milli-Q water and the stock concentration should be above 0.3 mg/mL.

Linear PCR templates obtained through a two-step overlap PCR and containing the T7 promoter and T7 terminator region can also be used. Linear PCR templates are less stable than plasmid templates; protective additives can be included, such as lambda gamS (Garamella et al., 2016).

### CFPS Reactions (Overnight)

For membrane proteins, a CECF configuration should be employed for the CFPS reaction to obtain higher expression yields because of the relatively low yields of membrane proteins when compared with those of cytosolic proteins. Both analytical- and preparative-scale reactions can be performed using different dialysis setups. Although we use the custom-made reactors shown in Figure 1, commercial dialysis devices can also work, providing that the reaction mixture (RM) and the feeding mixture outside (FM) are in good contact, such as when using a D-tube dialyzer (Novagen) combined with an Eppendorf tube, as shown in Figure 1. The RM: FM ratio should be between 1:14 and 1:20.

1. Calculate the volume required for each compound to perform a set number of reactions.
2. Prepare a common master mixture (RFM) containing the compounds required for both the RM and the FM (see Table 1). Combine all the compounds into one reaction tube.

**TABLE 1:** Common reagent mixture preparation (RFM) for CFPS with 1:16 ratio of RM: FM.

Compound	Stock	Final concentration	Volume^a^ [μl]
RCWMDE	16.67 mM	1 mM	1,020
Amino acid mix	4 mM	0.5 mM	2,337.50
Acetyl phosphate	1 M	20 mM	340
Phospho(enol) pyruvic acid	1 M	20 mM	340
75 × NTP mix	90 mM ATP	1.2 mM	226.7
	60 mM G/C/UTP	0.8 mM	
1,4 dithiothreitol	500 mM	2 mM	68
Folinic acid	10 mg/ml	0.1 mg/ml	170
Complete^®^ protease inhibitor	50×	1×	340
Hepes/EDTA buffer	24×	1 x	623.3
Mg(OAc)_2_	1 M	11.1 16, mM^b^	274
KOAc	4 M	110, 270, mM^b^	382.5
PEG 8000	40%	2%	850
NaN_3_	10%	0.05%	85
			Total: 7,057

*^*a*^Volumes are calculated for 1 ml RM and 16 ml FM = 17 ml RFM. ^*b*^Concentration is subject to optimization and might need to be adjusted for each individual target. Volumes are calculated for final total concentrations of Mg^2+^ of 16 mM, and K^+^ of 270 mM as additional amounts of 4.9 mM Mg^2+^ and 160 mM K^+^ are contributed by other compounds.*

3. Aliquot the amount of RFM required for the RM and complete the RM and FM with the remaining compounds (see Table 2).

**TABLE 2:** RM (1 ml) and FM (16 ml) preparation.

Compound	Stock	Final concentration	Volume
**FM:**			
RFM			6,642 μl
S30-C Puffer	1×	0.35×	5,600 μl
Amino acid mix	4 mM	0.5 mM	2,000 μl
MilliQ water			1,758 μl
			Total: 16 ml
**RM:**			
RFM			415 μl
Pyruvat kinase	10 mg/ml	0.04 mg/ml	4 μl
t-RNA (E. coli)	40 mg/ml	0.5 mg/ml	12.5 μl
T7RNAP	1.4 mg/ml	0.05 mg/ml	35,7 μl
Ribolock	40 U/μl	0.3 U/μl	7.5 μl
DNA template	0.2–0.5 mg/ml	0.015–0.03 mg/ml	60 μl
*E. coli* S30 extract	1×	0.35×	350 μl
MilliQ water			115.3 μl
			Total: 1 ml

4. Optimize the Mg^2+^ and plasmid concentrations to obtain the maximum yield and use them for the ensuing assay. The range of Mg^2+^ screening is normally between 10 and 20 mM, while the range of plasmid concentration screening is between 0 and 50 μg/mL.
5. For D-CFPS (Cell-free Protein Synthesis in the presence of detergents) screening reactions, detergents should be included in both the RM and FM. For L-CFPS (Cell-free Protein Synthesis in the presence of liposomes) reactions, liposomes should only be included in the RM.
6. Transfer the RM and FM aliquots into reaction containers and incubate overnight at 30°C with continuous agitation, either by shaking or rolling, depending on the reaction container set up. This is necessary to ensure efficient substance exchange between the RM and FM through the membrane. Shaking water baths or temperature-controlled cabinets with shaking plates (approximately 150–200 rpm) can be used.

### Sample Preparation for Functional Analysis

#### Protein Resolubilization (Optional)

Aquaporins can be expressed without the addition of detergents or lipids. However, the expressed aquaporins are in pellet form and an additional step of resolubilization with detergents is required.

1. After overnight expression, collect the RM and transfer into a new tube (this should be done carefully to collect as much of the RM as possible because the precipitated proteins might stick to the membrane). Resuspend *via* pipetting up and down before aspiration.
2. Collect the pellet fraction *via* centrifugation at 20,000 × *g* for 30 min at 4°C.
3. Wash the pellet twice with Buffer-P-B without DDM and finally resuspend with a mild detergent in an equal volume of RM. Different detergents should be screened since they might result in different resolubilization efficiencies. Our previous results showed that Fos-Chlorine-12, Fos-Chlorine-16, and LPPG display relatively high resolubilization percentages for mAQP4 M23 (Kai et al., 2010).
4. Incubate the sample mixture at 37°C for 30 min with vigorous shaking.
5. Centrifuge at 20,000 × *g* for 30 min and collect the super- natant for the next purification step. Analyze the supernatant and pellet by SDS–PAGE and immuno- detection to determine the resolubilization efficiency.

#### Protein Purification (3 H)

1. After overnight expression, collect the RMs from the D-CFPS reactions and transfer them into a new tube.
2. Centrifuge at 20,000 × *g* for 30 min at 4°C to collect the supernatant.
3. Combine the resulting supernatant or the supernatant resulting from the resolubilization step with nine volumes of Buffer P-B and mix with a proper amount of pre-equilibrated (with Buffer P-B) Ni-NTA resin slurry (in certain case Co^+^ was used instead of Ni^+^ for a better selectivity).
4. Incubate with gentle rolling at room temperature for 2 h and wash the resin with 10 volumes of Buffer P-W.
5. Elute with 3–5 volumes of Buffer P-E.
6. Analyze the results *via* SDS–PAGE or immunodetection.

#### Liposome Preparation (Overnight)

1. Solubilize the desired amount of lipid in chloroform or use presolubilized lipids in chloroform and transfer it into a round-bottom flask.
2. Form a thin lipid film *via* a vacuum rotary evaporator and leave the film under a complete vacuum overnight.
3. Reconstitute the dried lipid film in 1 mL of Buffer R to a final concentration of 20 mg/mL by vortexing for 15 min, forming multilamellar vesicles.
4. Extrude the resulting multilamellar vesicles 21 times through an Avanti Polar Lipids mini extruder holding a 200-nm Whatman polycarbonate membrane filter supported on both sides by two filter supports.
5. The resulting unilamellar liposome solutions can be used for the next reconstitution step.
6. Caution: Do not freeze the liposomes; use freshly prepared liposomes. For short storage, store the preformed liposomes at 4°C but not for longer than 1 week as liposomes are not stable and tend to fuse during storage.

#### Aquaporin Reconstitution (Overnight)

1. Make up the following solution by the sequential addition of 10 × Buffer R (final dilution 1×), followed 10% Triton X-100 (a final concentration of 4 mM), 20 mg/mL preformed liposomes (a final concentration of 4 mg/mL), and finally purified aquaporin (a final concentration of approximately 100 μg/mL). A total volume of approximately 1.6 mL is recommended.
2. Incubate the reconstitution mixture at room temperature with gentle shaking for 30 min.
3. Remove the detergent using SM-2 beads according to the manual.
4. Centrifuge the reconstitution mixture at 500,000 × *g* for 45 min.
5. Wash the pellet again with Buffer R and finally resuspend with 1.6 mL of Buffer R.

#### Sample Preparation for L-CFPS (Overnight)

1. Collect the RMs from the L-CFPS reactions after overnight incubation and transfer into new Eppendorf tubes.
2. Centrifuge at 18,000 × *g* for 30 min and collect the pellet, which contains a mixture of multilamellar lipids with incorporated aquaporin, as well as precipitated aquaporin.
3. Add an equal volume of Buffer R with either 0.42% (*w*/*v*) Triton X-100 or 1% (*w*/*v*) β-OG per 4 mg/mL lipids to solubilize the incorporated aquaporin with lipids. The remaining pellets are precipitated aquaporin.
4. Centrifuge at 18,000 × *g* for 10 min, collect the supernatants, and remove the detergent using either SM-2 beads or dialysis.
5. Follow the instructions in the manual for the SM-2 beads to remove Triton X-100. For the removal of β-OG, perform step-wise dialysis against Buffer R with 0.5, 0.25, 0.125, and 0% β-OG. Each dialysis step should run for 6 h at 4°C with constant stirring, except for the final step which should run overnight.
6. The reformed proteoliposomes are extruded again through a 200-nm membrane filter before measurement by stopped-flow spectrophotometry.

### Water Permeability Measurement Using a Stopped-Flow Spectrophotometer (1 H)

1. Determine the average diameter of the freshly prepared aquaporin-containing proteoliposomes by dynamic light scattering.
2. Measure the water permeability with a fixed light scattering angle (90 degrees) at 436 nm with temperature control.
3. When the system temperature is stable, the reconstituted proteoliposomes or reformed proteoliposomes from the L-CFPS reactions are mixed with an equal volume of hyperosmotic solution (Buffer R + 400 mM sucrose).
4. Data obtained from the spectrophotometer are fitted into an exponential rise equation. The initial shrinkage rate constant (*k*) is the average *k* vale of the best fitted exponential curve from 6–10 individual measurements.
5. Calculate the osmotic water permeability coefficients (*P*_f_) of the corresponding samples using the following equation [1]:

(1) $$P_{\text{f}}=\frac{k}{\left(S/V_{0}\right)\times V_{\text{w}}\,\text{×}\,\Delta_{\text{osm}}}$$

where, *S*/*V*_0_ is the vesicle’s initial surface-to-volume ratio; *V*_w_ represents the partial molar volume of water (18 cm^3^/mol); and Δ_*osm*_ is the osmotic driving force (200 mM if 400 mM sucrose is used for the hyperosmotic solution). The *S*/*V*_0_ is calculated by determining the diameters of the proteoliposomes using dynamic light scattering.

6. (Optional) A reversible mercury inhibitory assay can be performed to show aquaporin-facilitated water transport. Incubate the reconstituted proteoliposomes with 300 μM HgCl_2_ at the measuring temperature for 5 min. Then, follow steps 1–5 to obtain the *P*_f_ of the mercury-treated sample. The inhibition can be reversed by further treatment with 2 mM β-mercaptoethanol for 10 min.

## Anticipated Results

### Overexpression and Purification of Aquaporin From the CFPS System

**FIGURE 2:**
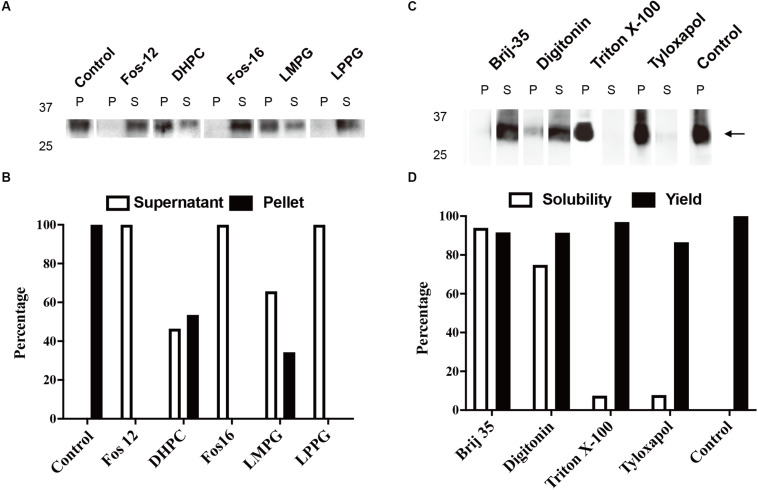
Cell-free protein synthesis (CFPS) of mAQP4 M23. **(A,B)** Detergent resolubilization screening of mAQP4 M23; **(C,D)** detergent screening of mAQP4 M23 for the D-CFPS (CFPS in the presence of detergent) reaction. **(A,C)** Immunoblotting using anti-His antibodies. **(B,D)** Quantification of the corresponding immunoblotting by densitometry. The detergent concentrations (indicated by *w*/*v* percentage) were: 1% Fos-Chlorine-12; 2% DHPC; 2% Fos-Chlorine-16; 2% LMPG {1-myristoyl-2-hydroxy-sn-glycero-3-(phospho-rac-[1-glycerol])}; 1% LPPG {1-palmitoyl-2-hydroxy-sn-glycero-3-(phospho-rac-[1-glycerol])}; 0.2% Brij-35; 0.4% digitonin; 0.1% Triton X-100; and 0.05% tyloxapol. The figure was adapted and reused from Kai et al. (2010), PloS ONE, under the terms of the Creative Commons Attribution License.

The typical yield of aquaporins produced using the above protocol was approximately 1 mg/mL of RM. Routine optimization should be performed to achieve the maximum yield, including optimization of Mg^2+^ and plasmid concentrations. Aquaporin can be produced as a precipitate and resolubilized posttranslationally using mild detergents and remain functional (Schwarz et al., 2007). As shown in Figures 2A,B, 1% (*w*/*v*) Fos-Chlorine-12, 2% (*w*/*v*) Fos-Chlorine-16, and 1% (*w*/*v*) LPPG showed almost 100% resolubilization efficiency. One advantage of this expression mode was that the pellet fraction was mainly obtained from newly expressed membrane proteins, which led to higher purity without the requirement for extensive purification steps. However, an additional resolubilization step was required, and it remained unclear whether the resolubilized aquaporin was fully or only partially functional. The D-CFPS expression mode provides a direct hydrophobic environment co-translationally and avoids the resolubilization step. Several detergents, such as Brij^®^35 and Brij^®^58, showed high efficiency in solubilizing newly expressed aquaporins. For instance, in 0.2% Brij^®^35, 90% of the mAQP4 M23 was expressed in the soluble fraction (Kai et al., 2010) (Figures 2C,D), while 1% Brij^®^S20 could support the soluble AqpZ expression (Yue et al., 2019). The optimum detergent might be target-dependent. Nevertheless, a general detergent selection criterion is that the detergent used should not decrease productivity, while still supporting a high solubility of the expressed protein in the CFPS environment.

Purification of resolubilized or D-CFPS-obtained aquaporin was performed through one affinity chromatography step and analyzed *via* SDS–PAGE. As indicated in the Methods section, during this step, a detergent exchange can be introduced because the detergents used in D-CFPS or resolubilization are often not suitable for the reconstitution step. First, dilution with the second detergent was performed to reduce the original detergent concentration. Then, a second washing step was carried out with a large volume of buffer containing the second detergent to further exchange and wash out the original detergent, leading to complete detergent exchange.

### L-CFPS Reactions

As stated in Section “Sample Preparation for L-CFPS (Overnight),” a mixture of lipids and incorporated aquaporins can be directly isolated with medium-speed centrifugation. Homogenous proteoliposome can be reformed through detergent resolubilization and removal, which should greatly accelerate the speed of sample preparation for functional analysis. The liposome concentration must be above 8 mg/mL in the RM to support higher incorporation (Roos et al., 2013; Yue et al., 2019). However, due to the volume limit of the CFPS system, it may be impossible to achieve such a high lipid concentration in the final RM. The solution is to either increase the liposome stock concentration or the concentration of the amino acid mixture (25 mM for each of the 20 amino acids).

### Water Permeability Measurements

**FIGURE 3:**
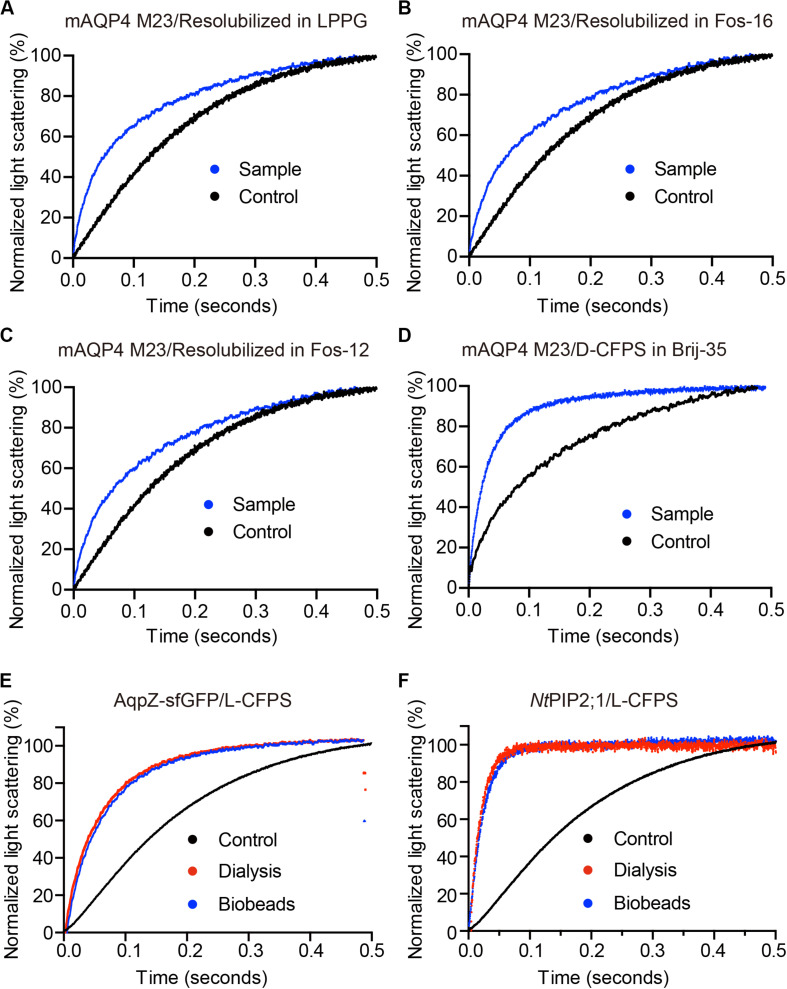
The water permeability of aquaporin-containing proteoliposomes prepared from different Cell-free Protein Synthesis (CFPS) modes. The scattered light intensities (arbitrary units) from each measurement obtained *via* stopped-flow spectrophotometry were normalized between 0 and 100 (the smallest value was defined as 0, the largest value as 100). Each curve shown in this figure represents an averaged and normalized curve of 6–10 individual measurements. The water transport experiments were performed at 10°C. **(A–C)** mAQP4 M23 expressed as pellet fractions and resolubilized in different detergents; **(D)** mAQP4 M23 expressed using D-CFPS (CFPS in the presence of detergent); **(E,F)** *Escherichia coli* AqpZ-sfGFP and *Nt*PIP2;1 (from *Nicotiana tabacum*) expressed using L-CFPS (CFPS in the presence of liposomes) and treated with different detergent removal methods to reform the proteoliposomes. Panels **(A–D)** were adapted and reused from Kai et al. (2010), PloS ONE, under the terms of the Creative Commons Attribution License; **(E,F)** were adapted and reused from Yue et al. (2019), Cells, under the terms of the Creative Commons Attribution License.

**FIGURE 4:**
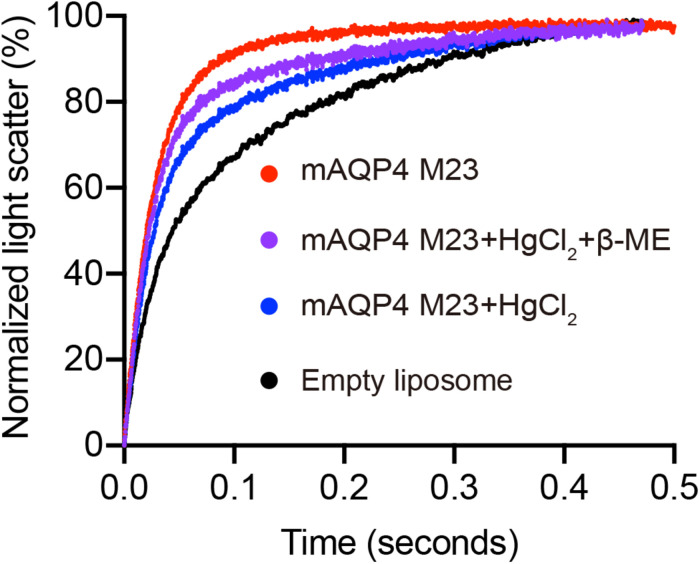
Reverse inhibitory assay to confirm mAQP4 M23-mediated water transport. Proteoliposomes containing mAQP4 M23 obtained by D-CFPS (Cell-free Protein Synthesis in the presence of detergent) were treated with 300 μM HgCl_2_ for 5 min at 23°C. For the recovery assay, 2 mM beta-mercaptoethanol was added, followed by incubation for 10 min. Empty liposomes with and without HgCl_2_ treatment were used as controls.

As shown in Figure 3, functional aquaporin proteoliposomes should show a faster increase in the light scattering signal than that of the empty, control liposomes when mixed with a hyperosmotic solution. The corresponding water permeability can be calculated *via* equation [1] depicted in Section “Water Permeability Measurement Using a Stopped-Flow Spectrophotometer (1 H).” As an example, the water permeabilities of the tested aquaporins (Figure 3) are summarized in Table 3. The binding of mercury ions to key cysteine residues inhibits the activities of most aquaporins (Savage and Stroud, 2007; Yukutake et al., 2008). Furthermore, a strong reducing reagent, such as β-mercaptoethanol, can be used to rescue this inhibition by competing with mercury for the binding to the key cysteine residues. Therefore, a reversible inhibitory assay can further confirm aquaporin-facilitated water transportation (Savage and Stroud, 2007; Yukutake et al., 2008; Kai et al., 2010). mAQP4 M23 was used as a model protein to test this reversible inhibitory assay (Figure 4). After treatment with mercury, the water permeability dropped from 160.01 ± 5.5 μm/s (*k* = 30.61 ± 1.05) to 94.9 ± 4.2 μm/s (*k* = 19.39 ± 0.81). However, the function of the blocked mAQP4 M23 could be rescued *via* β-mercaptoethanol treatment, resulting in a permeability of 137.5 ± 4.9 μm/s (*k* = 26.3 ± 0.94). The water permeability of empty, mercury-treated liposomes was set as control, displaying a water permeability of 50.3 ± 2.3 μm/s (*k* = 9.62 ± 0.45). No differences were detected between empty, non-treated liposomes and the mercury-treated samples (data not shown). The system temperature should be kept constant between individual measurements.

**TABLE 3:** Summary of calculated water permeability from samples shown in Figure 3.

Sample^a^	Diameter (nm)	*k*^b^	Pf (μm/s)b	Significant differences^c^	References
Control (A–D)	113	4.87 ± 0.31	25.5 ± 1.6	a	Kai et al., 2010
Sample A	113	9.71 ± 0.52	50.7 ± 2.7	b	Kai et al., 2010
Sample B	113	9.45 ± 0.38	49.4 ± 2.0	b	Kai et al., 2010
Sample C	113	10.41 ± 0.48	54.4 ± 2.5	b	Kai et al., 2010
Sample D	113	25.44 ± 1.07	133.1 ± 5.6	c	Kai et al., 2010
Control (E and F)	180	5.38 ± 0.16	44.88 ± 1.33	a	Yue et al., 2019
AqpZ-sGFP-Dialysis	210	15.49 ± 0.96	150.60 ± 9.33	b	Yue et al., 2019
AqpZ-sGFP-Biobeads	210	15.08 ± 0.62	146.61 ± 6.03	b	Yue et al., 2019
NtPIP2;1-Dialysis	195	54.23 ± 0.99	489.58 ± 8.94	c	Yue et al., 2019
NtPIP2;1-Biobeads	195	39.49 ± 0.59	356.51 ± 5.33	d	Yue et al., 2019

*^*a*^Control (A–D): empty liposomes shown in Figures 3A–D; Sample A–D: different mAQP4 M23 proteo-liposome samples shown in Figures 3A–D; Control (E and F): empty liposomes samples shown in Figures 3E,F. ^*b*^Both the rate constant and calculated P_*f*_ value of a corresponding sample were given as mean ± SD. ^*c*^Significant differences among different samples (indicated by different letters) were analyzed by one-way ANOVA followed by Tukey’s honest significant difference test (n = 6–10, P < 0.05).*

## Discussion

Although aquaporins can be successfully overexpressed in different host organisms such as *E. coli*, yeast, *X. laevis* oocytes, and insect cells, aquaporin sample preparation for functional and structural analysis remains a great challenge. An alternative recombinant protein production method, namely, the CFPS system, shows advantages over cellular expression systems with regard to membrane proteins (Rues et al., 2018). The CFPS system, which is devoid of living host cells, provides a flexible co-translation environment for the production of aquaporin, without the concern of maintaining living host cells. In addition, processes such as aquaporin extraction from the membrane of host cells or refolding of inclusion bodies can be completely avoided. Instead, the CFPS system allows the screening of translational conditions to achieve better folding. This is normally done through the introduction of additives, such as detergents or lipids, directly into the CFPS reaction mixture.

In this protocol, we have provided a systematic strategy for aquaporin sample preparation. This strategy is suitable for water permeability measurements and other applications that require functional aquaporin either in detergent micelles or liposomes, i.e., water filtration membranes (Wang et al., 2015). Three different expression modes were introduced: (i) CFPS without the addition of hydrophobic reagents; (ii) the D-CFPS mode; and (iii) the L-CFPS mode. The first expression mode is suitable for the first round of yield optimization, such as optimization of Mg^2+^ and plasmid concentrations. As indicated in the Results section, the membrane protein pellet was often purer than that obtained by D-CFPS. Although the pellet fractions could not always be functionally resolubilized, the D-CFPS mode provided co-translational solubilization of aquaporin, offering a direct hydrophobic environment for the folding of the target proteins. The D-CFPS mode was designed to obtain large quantities of aquaporins. The L-CFPS setup is the most efficient for the fast characterization of the functionality of specific aquaporins based on the proteoliposome assay. First, no additional purification step is needed. Second, the liposome lipid biolayer is a better mimic of the biological membrane than detergent micelles for aquaporin folding. However, after overnight expression, the resulting proteoliposomes were fused and formed a multilamellar lipid clot. Even at low speeds, centrifugation could still pellet all the lipid fractions (Yue et al., 2019). Therefore, it was not possible to directly use these samples for water permeability assays, as homogenously sized proteoliposomes are required for this process. Although it has been reported that low centrifugation can still provide proteoliposome suspensions that can be re-extruded through a filter membrane to reform homogenously sized samples (Hovijitra et al., 2009), this was not possible in our hands, which may have been due to several reasons. For instance, high Mg^2+^ and PEG concentrations may have promoted liposome fusion, or the relatively long reaction times and high yields may have resulted in additional protein precipitation before their incorporation into liposomes. Consequently, a large amount of non-incorporated aquaporin was present in the pellet fraction derived from the L-CFPS reaction. The key step involving mild detergent resolubilization provided a solution for the separation of the precipitated protein and the incorporated protein–lipid mixture. Homogenous aquaporin proteoliposome samples can be reformed following a detergent removal step. Both SM-2 beads and dialysis can then be used to remove the detergent. However, this may lead to small differences in the final water permeability of individual aquaporins, depending on which method is used. With the above-described methods, we successfully characterized mAQP4 M23 from the mouse (Kai et al., 2010), AqpZ from *E. coli*, *Nt*PIP2;1 from *Nicotiana tabacum* (Kai and Kaldenhoff, 2014; Yue et al., 2019), human AQP3 (Muller-Lucks et al., 2013), *Pf*AQP from *Plasmodium falciparum* (Muller-Lucks et al., 2012), and AqpB from *Dictyostelium discoideum* (von Bulow et al., 2012). In particular, the mercury inhibitory assay showed that reconstituted mAQP4 M23 was inhibited by mercury, which was different from AQP4 expressed in cell membranes. In line with the results of a previous study (Yukutake et al., 2008) it is reasonable to hypothesize that the reconstitution conditions followed in our study leads to bidirectional insertion of mAQP4 M23, exposing the critical cysteine (C178, loop D) to the outside of the liposome, and therefore accessible to the mercury ion (Kai et al., 2010). In addition, through the same sample preparation procedure, *Nt*AQP1 produced through D-CFPS could be applied to a CO_2_ permeability assay using a modified black lipid membrane system (Kai and Kaldenhoff, 2014). Furthermore, this protocol can be easily adapted and modified to determine other aquaporin channel activities if the functional assay, such as that for glycerol, is based on proteoliposomes (Hovijitra et al., 2009). Because the CFPS system introduced here was in a reduced environment, the system must be adjusted through the introduction of a redox buffer, as well as DsbC, to allow the formation of correct disulfide bridges in specific aquaporins (Hachez et al., 2013; Matsuda et al., 2013; Rues et al., 2018; Dopp and Reuel, 2020).

## Conclusion

We described a CFPS system-based sample preparation approach for the functional analysis of aquaporins. Three expression modes were applied that were suitable for different aquaporins. In particular, the L-CFPS mode approach is a fast, efficient, and convenient method for the functional characterization of aquaporins. Finally, this protocol can also be adapted for the preparation of samples for use in plenary or vesicular lipid-based functional assays involving other channel proteins or transporters.

## Data Availability Statement

All datasets generated for this study are included in the article/supplementary material.

## Author Contributions

KY and LK performed the experiments. All authors contributed to the conceptualization and writing and editing of this manuscript.

## Conflict of Interest

The authors declare that the research was conducted in the absence of any commercial or financial relationships that could be construed as a potential conflict of interest.
